# Caustic Pommade (Baummés)

**Published:** 1844-10-19

**Authors:** 


Caustic Pommade (Baummés).—Eight grammes of lard, one gramme of powdered savine, one gramme of alum, one gramme of colomel. Used in frictions of vegetations, the most fungous and vascular especially, become inflamed, suppurate, shrink up, and come away by shreds.—*Ibid.*

Mr. Weninger, of Vienna, has published a case where vaccination was performed on a child eight months old, in July, 1837, and did not become developed till July, 1840 thus remaining latent for three years!—*Ibid.*

